# Quantitative Profiling of Feruloylated Arabinoxylan Side-Chains from Graminaceous Cell Walls

**DOI:** 10.3389/fpls.2015.01249

**Published:** 2016-01-14

**Authors:** Rachel R. Schendel, Marleen R. Meyer, Mirko Bunzel

**Affiliations:** Department of Food Chemistry and Phytochemistry, Institute of Applied Biosciences, Karlsruhe Institute of TechnologyKarlsruhe, Germany

**Keywords:** plant cell walls, cereal grains, feruloylated oligosaccharides, arabinoxylans, screening, ferulic acid, arabinoxylan side-chains

## Abstract

Graminaceous arabinoxylans are distinguished by decoration with feruloylated monosaccharidic and oligosaccharidic side-chains. Although it is hypothesized that structural complexity and abundance of these feruloylated arabinoxylan side-chains may contribute, among other factors, to resistance of plant cell walls to enzymatic degradation, quantitative profiling approaches for these structural units in plant cell wall materials have not been described yet. Here we report the development and application of a rapid and robust method enabling the quantitative comparison of feruloylated side-chain profiles in cell wall materials following mildly acidic hydrolysis, C18-solid phase extraction (SPE), reduction under aprotic conditions, and liquid chromatography with diode-array detection/mass spectrometry (LC-DAD/MS) separation and detection. The method was applied to the insoluble fiber/cell wall materials isolated from 12 whole grains: wild rice (*Zizania aquatica* L.), long-grain brown rice (*Oryza sativa* L.), rye (*Secale cereale* L.), kamut (*Triticum turanicum* Jakubz.), wheat (*Triticum aestivum* L.), spelt (*Triticum spelta* L.), intermediate wheatgrass (*Thinopyrum intermedium*), maize (*Zea mays* L.), popcorn (*Zea mays* L. var. *everta*), oat (*Avena sativa* L.) (dehulled), barley (*Hordeum vulgare* L.) (dehulled), and proso millet (*Panicum miliaceum* L.). Between 51 and 96% of the total esterified monomeric ferulates were represented in the quantified compounds captured in the feruloylated side-chain profiles, which confirms the significance of these structures to the global arabinoxylan structure in terms of quantity. The method provided new structural insights into cereal grain arabinoxylans, in particular, that the structural moiety α-l-galactopyranosyl-(1→2)-β-d-xylopyranosyl-(1→2)-5-*O*-*trans*-feruloyl-l-arabinofuranose (**FAXG**), which had previously only been described in maize, is ubiquitous to cereal grains.

## Introduction

Ferulic acid is found ester-linked to arabinoxylans in the primary and secondary cell walls of grasses. These feruloylated arabinoxylans are a distinguishing feature of grass cell walls and represent their principal hemicellulosic component, making up 20–40 and 40–50%, respectively, of primary and secondary grass cell walls on a dry weight basis (Vogel, 2008). However, feruloylated arabinoxylans are not limited to the Poaceae family, but are found in the primary cell walls of all commelinid monocotyledons (Harris and Trethewey, 2010).

All feruloylated arabinoxylans are based on a (1→4)-linked β-d-xylopyranosyl backbone structure decorated with various substituents. α-l-arabinofuranose dominates the substitution landscape as either a mono- and/or di-substituent at the xylopyranosyl *O*-3 and/or *O*-2 positions, but additional backbone decorations such as glucuronic acid, its 4-*O*-methyl derivative, or acetyl are also observed, particularly in maize and sorghum arabinoxylans (Verbruggen et al., 1995; Huisman et al., 2000; Kabel et al., 2002; Appeldoorn et al., 2010, 2013). The acylation of a portion of the arabinose substituents at their *O*-5 position by phenolic acids, particularly *trans*-ferulic acid, is a key feature of arabinoxylans. Some of the ferulate residues undergo free radical-induced oxidative coupling to form ferulate dimers and higher oligomers, thus creating inter- and intramolecular cross-links between arabinoxylan chains; ferulate and diferulate coupling with lignin monomers and oligomers also results in arabinoxylan-lignin cross-linkages (Ralph et al., 1994, 1995; Saulnier et al., 1999; Allerdings et al., 2005; Bunzel et al., 2008). Arabinoxylan cross-linking is important for both plant physiological processes and utilization of graminaceous cell walls. In the plant, cross-linking is suggested to be involved in cessation of cell extension by increasing the cell wall's stiffness (Kamisaka et al., 1990), thus also improving plants' disease and insect resistance (de O Buanafina, 2009; de O Buanafina and Fescemyer, 2012). Increased ferulate cross-linking in plant materials enhances their resistance to enzymatic degradation, thus decreasing the digestibility of forages for livestock and impeding breakdown of forage and grain byproduct materials during second-generation biofuel production (Grabber et al., 1998; Chundawat et al., 2011; Jung et al., 2012).

In insoluble fiber from cereal grains, the molar percentage of total diferulates as percentage of the sum of monomeric and dimeric ferulates ranges between 26% (spelt) and 45% (oats) (Bunzel, 2001). Therefore, a substantial portion of the total ferulates in grasses remains in monomeric form as feruloylated side-chains. In addition to the ubiquitous 5-*O*-*trans*-feruloyl-l--arabinofuranose (**FA**) structure, some grasses produce more complex, oligosaccharidic feruloylated side-chains. The feruloylated disaccharide, β-d-xylopyranosyl- (1→2)- 5-*O*-(*trans*-feruloyl)-l-arabinofuranose (**FAX**) has been identified in the leaves of various grasses (Wende and Fry, 1997) as well as rye, maize, wild rice, and intermediate wheat grass grains (Saulnier et al., 1995; Bunzel et al., 2002; Steinhart and Bunzel, 2003; Schendel et al., 2015). In maize grain, substantial amounts of the feruloylated trisaccharide, α-l-galactopyranosyl- (1→2)- β-d-xylopyranosyl- (1→2)-5-*O*-*trans*- feruloyl-l-arabinofuranose (**FAXG**), and smaller amounts of two feruloylated tetrasaccharides, α-d-galactopyranosyl- (1→3)- α-l-galactopyranosyl- (1→2)- β-d-xylopyranosyl- (1→2)- 5-*O*-*trans*-feruloyl-l-arabinofuranose (**FAXGG**), and α-d-xylopyranosyl- (1→3)-α-l-galactopyranosyl-(1→2)-β-d-xylopyranosyl-(1→2)- 5-*O*-*trans*-feruloyl-l-arabinofuranose (**FAXGX**) were detected following preparative isolation (Saulnier et al., 1995; Allerdings et al., 2006).

Although the effects of these monomeric ferulate side-chains have not been as thoroughly researched as ferulate crosslinking, both increased appearance and complexity of feruloylated side-chains are hypothesized to reduce enzymatic digestibility and slow fermentation of feruloylated arabinoxylans (Yang et al., 2013; de Vries et al., 2014; Snelders et al., 2014), which has implications for plant protection mechanisms against pathogens, the prebiotic and (potential) antioxidative human health benefits of dietary fibers from different cereal grains, livestock nutrition, and biofuel production. However, to date, a direct quantitative comparison of the feruloylated side-chain profiles from different grain or forage materials has not been performed, and estimations of profile complexity have been based on results from time-consuming preparative isolations (Saulnier et al., 1995; Bunzel et al., 2002; Allerdings et al., 2006). We have, therefore, developed a liquid chromatography with diode-array detection/mass spectrometry (LC-DAD/MS) screening method enabling quantitative comparison of the feruloylated side-chain profiles of plant cell wall materials following mildly acidic hydrolysis, C18-solid phase extraction (SPE), and reduction.

## Materials and methods

### Chemicals

Trifluoroacetic acid (TFA) and sodium borohydride were from Sigma (Seelze, Germany). *Trans*-ferulic acid was from Fluka (Buchs, Switzerland). DMSO was from Roth (Karlsruhe, Germany). All chromatography solvents (MS grade) were from VWR (Bruchsal, Germany). Water was deionized and filtered through a Milli-Q Reference water purification system (Merck Millipore, Billerica, Massachusetts, USA).

### Plant materials and enzymes

Cellulose was from Roth, xylans (from beechwood) were purchased from Sigma. Maize (*Zea mays* L.) middlings were a gift from Cornexo GmbH (Freimersheim, Germany). Intermediate wheatgrass (*Thinopyrum intermedium*) grain was kindly shared by Dr. Lee DeHaan from The Land Institute, Salina, Kansas, USA. Wholegrain maize flour (*Zea mays* L.) was a gift from Mühle Beck (Keltern, Germany). Wild rice (*Zizania aquatica* L.), long-grain brown rice (*Oryza sativa* L.), rye (*Secale cereale* L.), kamut (*Triticum turanicum* Jakubz.), wheat (*Triticum aestivum* L.), spelt (*Triticum spelta* L.), popcorn (*Zea mays* L. var. *everta*), oat (*Avena sativa* L.) (dehulled), barley (*Hordeum vulgare* L.) (dehulled), and proso millet (*Panicum miliaceum* L.) whole grains were purchased from local grocery stores. Thermostable α-amylase (Termamyl 120 L), amyloglucosidase (AMG 300 L), and protease (Alcalase 1.5 MG Type FG) for preparative fiber isolation were kind gifts from Novozymes (Bagsvaerd, Denmark).

### General

SPE cartridges (Chromabond C18, 500 mg, 3 mL) were from Macherey-Nagel (Düren, Germany). Amberlite XAD-2 column material was from Supelco (Bellafonte, Pennsylvania, USA). Sephadex LH-20 material was from Sigma.

### Isolation of insoluble fiber material

All grain materials were ground to <0.5 mm (IKA MF10 Basic mill, Staufen, Germany) and partially de-fatted by washing with acetone. Insoluble fiber was isolated in triplicate (except for maize middlings, where the isolated material was pooled into one sample) from each of the grain materials in preparative quantities as described by Bunzel et al. (2001) with minor modifications. Briefly, for each triplicate sample 20 g of defatted flour was mixed with 200 mL of sodium phosphate buffer (0.08 M, pH 6.2) and 1.5 mL of thermostable α-amylase in a 500 mL Erlenmeyer flask and incubated at 92°C for 20 min (flasks were swirled every 5 min). The suspension was cooled to room temperature, adjusted to pH 7.5 with 0.275 M aqueous NaOH solution, and incubated with protease (700 μL) for 30 min at 60°C in a shaking water bath. The suspension was cooled to room temperature, adjusted to pH 4.5 with 0.325 M aqueous HCl solution, and incubated with 700 μL of amyloglucosidase for 30 min at 60°C in a shaking water bath. The warm suspensions were centrifuged (5 min, 5000 × *g*), the supernatant was decanted, and the insoluble residue was washed with warm water (60°C, 2 × 100 mL, 1 × 50 mL), ethanol (1 × 100 mL, 1 × 50 mL), and acetone (1 × 100 mL, 1 × 50 mL). Washed residues were dried in a vacuum oven at 70°C for 20 h and stored in a desiccator until use. Each triplicate was individually corrected for residual protein and ash. Protein content was determined as total Kjeldahl nitrogen (N × 6.25) using the colorimetric method developed by Willis et al. (1996). Ash content was determined by incineration at 525°C for 11 h.

### Preparation of standard compounds FA, FAX, and FAXG

**Figure 1:**
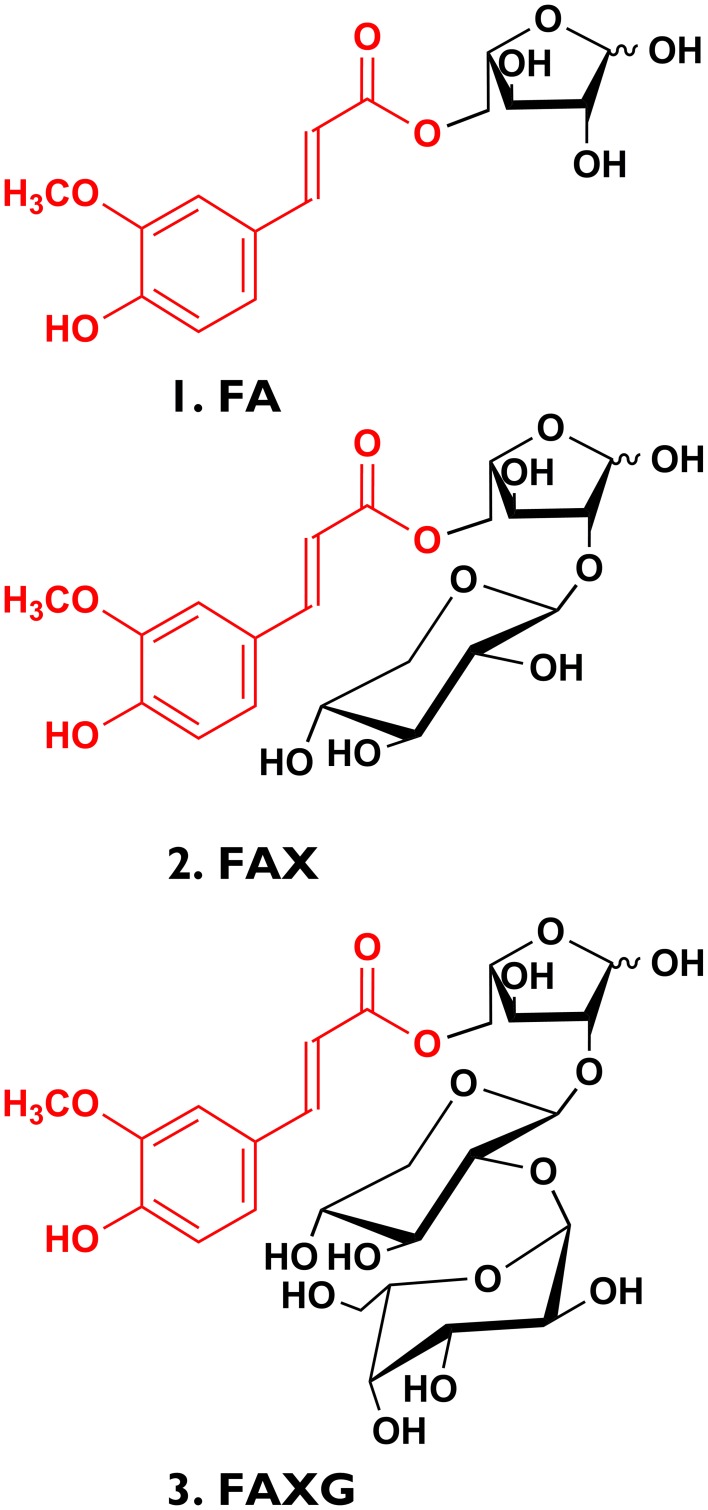
**Feruloylated arabinoxylan side-chain standard compounds isolated from insoluble maize fiber in preparative quantities**. **(1) FA**, 5-*O*-*trans*-feruloyl-l-arabinofuranose; **(2) FAX**, β-d-xylopyranosyl- (1→2)-5-*O*-(*trans*-feruloyl)- l-arabinofuranose; **(3) FAXG**, α-l- galactopyranosyl-(1→2)- β-d-xylopyranosyl- (1→2)- 5-*O*-*trans*-feruloyl-l-arabinofuranose.

**FA**, **FAX**, and **FAXG** (see Figure 1) were isolated in preparative quantities from insoluble fiber from maize middlings using a modified version of the method from Allerdings et al. (2006). Briefly, 20 g of insoluble fiber was hydrolyzed under mildly acidic conditions [400 mL of 0.05 M trifluoroacetic acid, 3 h, 100°C]. The supernatant containing the released feruloylated oligosaccharides was applied to a glass column (25 × 4 cm) filled with Amberlite XAD-2 material. Non-feruloylated oligosaccharides were washed from the column with water (600 mL), and feruloylated oligosaccharides were eluted with MeOH-H_2_O (600 mL, 50/50, v/v). A moderate elution rate was maintained (3–5 mL/min). The MeOH-H_2_O eluate, which contains the majority of the feruloylated oligosaccharides, was concentrated under vacuum with a rotary evaporator to 10 mL. This concentrate was directly injected (injection loop volume of 10 mL) on to a Sephadex LH-20 gel chromatography column (100 × 2.5 cm) using 100% water as the eluent (1 mL/min, Jasco 880 pump, Easton, Maryland, USA). The column eluate was monitored using UV absorption (355 nm, K2600 UV detector, Knauer, Berlin Germany), and fractions were collected at 6-min intervals (FC 204 fraction collector, Gilson, Middleton, Wisconsin, USA), pooled based on detected peaks, and concentrated under vacuum with a rotary evaporator. The pooled fractions corresponding to **FA**, **FAX**, and **FAXG** (Sephadex LH-20 retention times of 2100–2600 min, 1550–1830 min, and 950–1120 min, respectively) were purified on a preparative RP-C18 column (250 × 25 mm, 5 μm, 100 Å, Phenomenex, Aschaffenburg, Germany) using a preparative HPLC system consisting of two LC-8A pumps and a SPD-20A UV-Vis detector (325 nm, Shimadzu, Duisburg, Germany). The following binary gradient was used (eluent A = water + 0.1% formic acid, eluent B = MeOH): initially 75% A, 25% B; linear over 5 min to 30% B, linear over 20 min to 38% B, linear over 5 min to 100% B, hold for two min, linear over 1 min to 25% B, and re-equilibration for 7 min. Flow rate was 8 mL/min, and fractions were collected manually. Purity of the isolated standard compounds was confirmed by 1D- (^1^H) and 2D-NMR (^1^H-^1^H-COSY, HSQC, and HMBC) experiments at 298 K using an Ascend 500 MHz spectrometer (Bruker, Rheinstetten, Germany) with a Prodigy cryoprobe. For NMR analysis, 5–10 mg of samples were deuterium-exchanged twice and dissolved in D_2_O, with acetone (0.5 μL) as reference (δ^1^H 2.22, δ^13^C 30.89; (Gottlieb et al., 1997)). Standard compounds were dried by lyophilization and stored at −20°C.

### Quantitative comparison of feruloylated side-chain profiles of plant cell wall materials

#### Selective release of side-chains by mildly acidic hydrolysis

Insoluble fiber materials were hydrolyzed in triplicate under the conditions described by Saulnier et al. (1995) for optimal side-chain release. Insoluble fiber material (200 mg; 100 mg may also be used for samples with high side-chain concentrations, for example, maize-based materials) was weighed into a 15-mL Pyrex tube, suspended in 5 mL of 50 mM TFA, capped, vortexed, and incubated for 2 h at 100°C while protected from light. The samples were cooled on ice and centrifuged (10 min, 2000 × *g*). An aliquot (3 mL for most samples, 3.5 mL for spelt, 1.5 mL for maize and popcorn) of the supernatant was cleaned up using C18 SPE cartridges. The cartridges were washed with MeOH (6 mL) and re-conditioned with water (6 mL) before application of the hydrolysate aliquot. The loaded cartridge was washed with water (6 mL), and the feruloylated side-chains were eluted with MeOH (6 mL) into a small Pyrex tube (15 mL). The solutions were evaporated with a rotary evaporator (maximum water bath temperature of 45°C); residual water was removed in a vacuum drying oven at 40°C.

#### Reduction

The isolated side-chains in the dried residues were reduced to their corresponding sugar alcohols with NaBH_4_ in DMSO (2 mL, 30 mg NaBH_4_/mL DMSO, solution freshly prepared by sonicating in a sealed Pyrex glass at 45°C) for 18 h at 30°C with stirring [in tightly sealed small Pyrex tubes (15 mL)]. Samples were protected from light to prevent *cis*/*trans* isomerization. The reaction was stopped with the rapid dropwise addition of 2 mL of 1 M aqueous HCl. (Caution: plant hydrolysate samples may foam significantly at this step). Samples were directly analyzed with LC-DAD/MS or, if necessary, diluted with 50/50 DMSO/H_2_O (v/v).

#### LC-DAD/MS analysis

The reduced samples were analyzed on a Thermo Scientific (Waltham, Massachusetts, USA) system consisting of a Finnigan Surveyor Plus autosampler, pump, and photodiode array detector, and an LXQ-Triple Quad ion trap MS^n^ detector. 25 μL of the sample was separated at room temperature on a Luna C18 column (250 × 4.6 mm, 5 μM particle size) from Phenomenex (Aschaffenburg, Germany) using a 0.5 mL/min flow rate and a binary gradient made up of water with 0.1% formic acid (eluent A) and acetonitrile with 0.1% formic acid (eluent B). The initial gradient conditions were 95% A, 5% B; hold for 4 min; B from 5 to 10% in 4 min; hold for 2 min; B from 10 to 16% in 4 min; hold for 9 min; B from 16 to 50% in 10 min; B from 50 to 95% in 1 min; hold for 2 min; B from 95 to 5% in 1 min; re-equilibrate for 5 min. The eluate was monitored at 325 nm on the photodiode array detector. Ionization for the MS system was performed in the ESI positive mode, capillary temperature was 350°C, spray voltage was 4 kV, and capillary voltage was 47 V.

#### Quantification of FA, FAX, FAXG, and free ferulic acid

External calibration curves were prepared in triplicate for **FA**, **FAX**, and **FAXG** by weighing in appropriate amounts of the dried standard compounds, dissolving in MeOH and diluting as necessary. Aliquots of increasing volume were pipetted from the respective triplicate stock solutions into 15-mL Pyrex tubes to create equidistant, five-point calibration curves, dried down, reduced using the method described above (except the volumes of the NaBH_4_ in DMSO and 1M HCl solutions were both reduced to 1 mL), and analyzed using the LC-DAD/MS method (quantification performed via UV-absorbance at 325 nm; ESI-MS and MS/MS used for confirmation of peak identity) described above. The concentration range of the **FA** calibration curve was 10–260 μM, and **FAX** and **FAXG** were calibrated from 6 to 26 μM. Free ferulic acid released during the TFA hydrolysis was quantified by preparing stock solutions of *trans*-ferulic acid in triplicate in MeOH/H_2_O (50/50, v/v) and diluting appropriately to create six-point calibration curves (calibration range: 6–25 μM).

#### Method validation

Limits of detection (LOD) and limits of quantification (LOQ) were calculated as signal-to-noise ratios of 3:1 and 9:1, respectively. Linearity was assessed by inspecting the residual plots and correlation coefficients. Robustness of the quantification method for **FA**, **FAX**, and **FAXG** was tested by preparing six separate calibration curves (each originating from separate stock solutions) for each compound at different times (three calibration curves for each compound were prepared at the same time as a set, with four months' time between the first and second set). Percent recovery from the SPE and reduction steps in the method was calculated by drying down aliquots of **FA**, **FAX**, and **FAXG** in triplicate. The residues were dissolved in 100 μL of DMSO, and mixed with 3 mL of hydrolysate from a 1:1 (w:w) mixture of cellulose and beechwood xylan subjected to the mildly acidic degradation method described. This mixture was then carried through SPE-cleanup, reduction, and LC-DAD/MS analysis.

### Total ester-linked *trans*-ferulic acid analysis

The total ester-linked *trans*-ferulic acid in all insoluble fiber materials except for intermediate wheatgrass was determined according to Dobberstein and Bunzel (2010), with slight modifications. The method used for quantification of *trans*-ferulic acid in intermediate wheat grass was very similar (see (Schendel et al., 2015) for an exact description). 25–50 mg (with the exception of insoluble fiber from oats, where 100 mg was used) of insoluble fiber material from each of the sample triplicates was weighed in, and *ortho*-coumaric acid (5 mM stock solution, prepared in MeOH/H_2_O (50/50, v/v)) was added as an internal standard in the appropriate volume to create a concentration of 250 μM in the end samples. The samples were stirred with 5 mL of 2 M NaOH for 18 h at room temperature in the dark, acidified to pH 2 with ≈1 mL 37% HCl, and extracted with diethyl ether (3 ×, 8, 6, and 4 mL). The ether volumes were combined, dried under N_2_, dissolved in MeOH/H_2_O (50/50, v/v), and analyzed with a Shimadzu (Duisburg, Germany) HPLC-DAD system consisting of three LC-20AT pumps and a DGU-20A3 degasser, SIL-20AC autosampler, CTO-20AC column oven, and SPD-M20A diode array detector. Samples were separated on a Phenomenex Luna phenyl-hexyl column (250 × 4.6 mm, 5 μM particle size) using a tertiary gradient made up of 1 mM TFA in water (eluent A); acetonitrile/1 mM TFA in water, 90/10, v/v (eluent B); and MeOH/1 mM TFA in water, 90/10, v/v (eluent C). The gradient was performed as follows: initial conditions, 87% A, 13% B; hold for 11 min; B from 13 to 15% in 12 min; B from 15 to 16% in 5 min; B from 16 to 50% and C from 0 to 25% in 4 min; B from 50 to 13%, and C from 25 to 0% in 1 min; followed by a re-equilibration step. Injection volume was 10 μL, and flow rate was 1 mL/min. Quantification was performed at 325 nm with a linear, 12-point internal calibration curve prepared for *trans*-ferulic acid (concentration range: 5–2500 μM), using *ortho*-coumaric acid as the internal standard (concentration: 250 μM).

## Results

### Quantitative screening of feruloylated side-chain profiles: reduction method development

**Figure 2:**
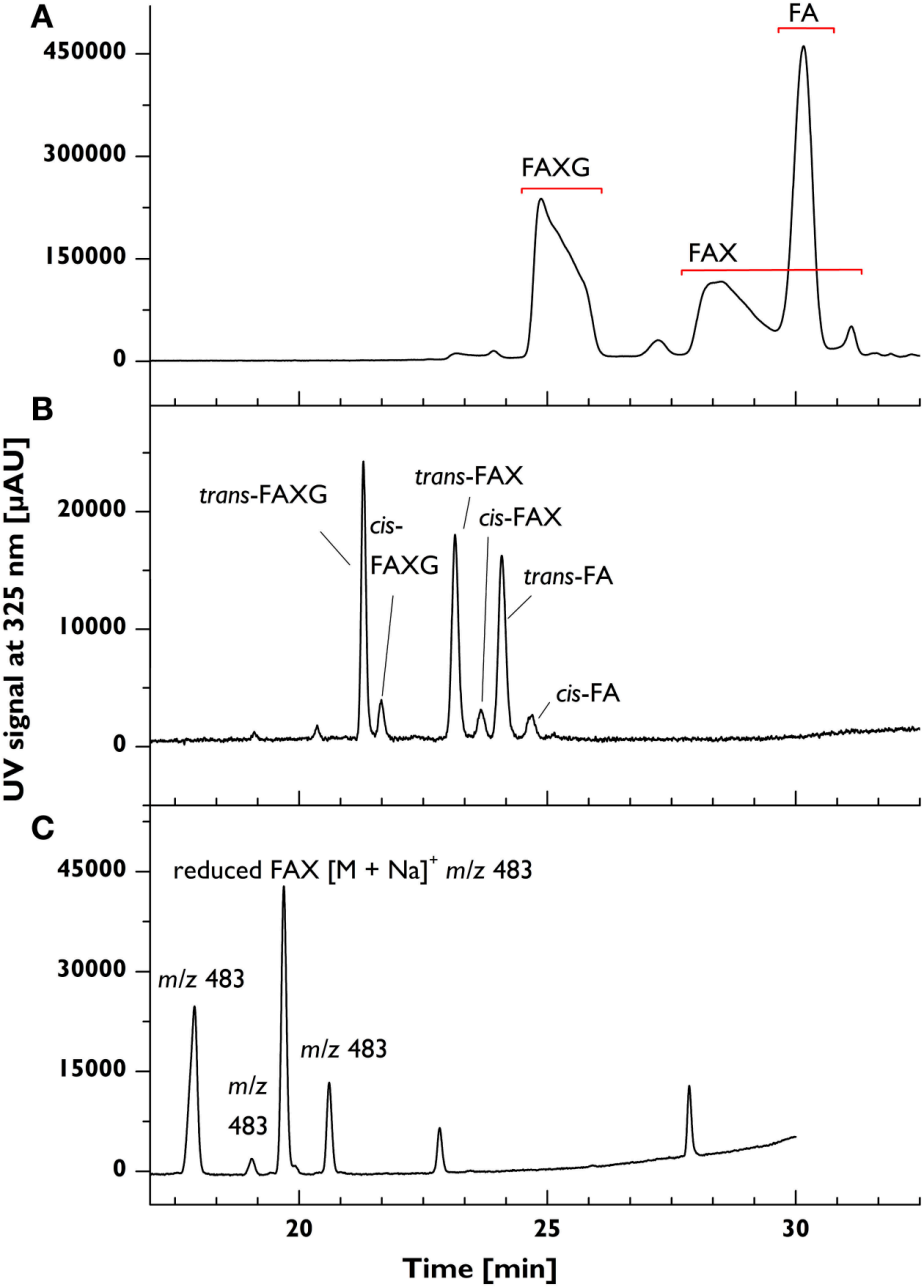
**Chromatographic peak forms of feruloylated side-chain standard compounds before and after reduction in various solvents**. [Top] **(A)** Broad, tailing, split peaks resulting from native (unreduced) feruloylated side-chain standard compounds. [Middle] **(B)** Narrow, symmetrical peaks resulting from reduction in aprotic solvent (DMSO) of feruloylated side-chain standard compounds to their respective sugar alcohols. [Bottom] **(C)** Lobry de Bruyn–van Ekenstein transformation of **FAX** in protic solvent (here: EtOH) during reduction. *Chromatographic conditions described in Materials and Methods; bottom chromatogram was obtained using a different gradient*. **FA**, 5-*O*-*trans*-feruloyl-l-arabinofuranose; **FAX**, β-d-xylopyranosyl- (1→2)-5-*O*-(*trans*-feruloyl)- l-arabinofuranose; **FAXG**, α-l- galactopyranosyl-(1→2)- β-d-xylopyranosyl- (1→2)- 5-*O*-*trans*-feruloyl-l-arabinofuranose; *m/z*, mass-to-charge ratio.

The reducing sugar moiety of the native standard compounds rules out their chromatographic separation under the primarily aqueous conditions applied in C18-based HPLC, where on-column mutarotation between the reducing sugar's α- and β-anomers, which differ slightly in their retention factors, produces broad, tailing, split peaks (see Figure 2A). This was especially true for **FAX**, which spawned a peak over 4 min wide. Reduction of the standard compounds to sugar alcohols with sodium borohydride in the aprotic solvent DMSO resulted in sharp, baseline-separated peaks, including separation of the *cis*- and *trans*-isomers of each compound (see Figure 2B, which depicts a chromatogram from a mixture of *cis*- and *trans*-isomers).

Several challenges were encountered during development of the reduction method. The first, the appearance of additional peaks in the chromatogram with the same mass-to-charge ratio (*m/z*) as the reduced standard compounds (see Figure 2C), arose when the reduction was performed in a protic solvent (for example, MeOH, EtOH, or DMSO-H_2_O mixtures). This behavior was attributed to a base-catalyzed Lobry de Bruyn-van Ekenstein transformation of the reducing arabinose to other sugars via an enediol reaction intermediate. The use of DMSO, an aprotic solvent, resulted in one chromatographic peak per standard compound. Nevertheless, care must still be taken during the reduction to avoid accidentally introducing water into the system. Both DMSO and sodium borohydride are strongly hygroscopic, so the reducing solution should be freshly prepared in a sealed container and the individual samples should be tightly sealed during reduction. Additionally, the HCl workup at the end of reduction should be performed as quickly as feasible without allowing the sample to foam over: excessive time spent in completing the acid addition creates a temporarily alkaline, protic environment, which facilitates the transformation reaction.

A second, sporadically occurring, hurdle in reduction method development was incomplete reduction, especially with the standard compound **FA**, over a range of reduction times and sodium borohydride concentrations. The final reduction conditions were chosen because the incidence of incomplete reduction was decreased, although not entirely eliminated. However, when occurring, the maximum amount of unreduced **FA** represented only ≈5% of the total **FA** in the samples and may, therefore, be neglected.

### Method validation results

**Figure 3:**
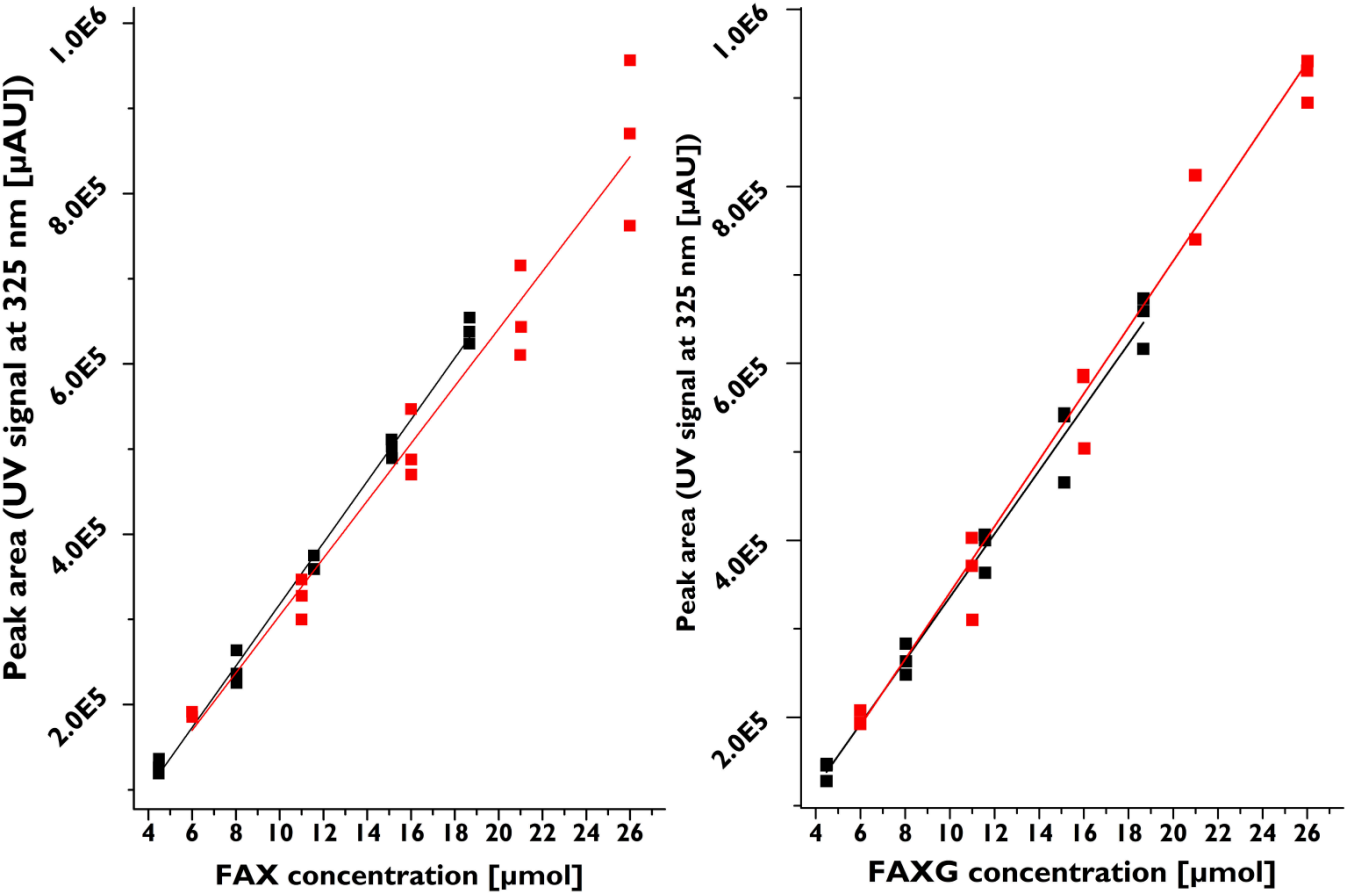
**Robustness of feruloylated side-chains quantification method as evidenced by linear correlation of six separate calibration curves prepared and analyzed four months apart**. Each calibration curve was prepared from individual stock solutions. Black signals, first three calibration curves, concentration range 4.5–18.6 μM; red signals, three calibration curves prepared four months later, concentration range 6.0–26.0 μM. **FAX**, β-d-xylopyranosyl-(1→2)-5-*O*-(*trans*-feruloyl)-l-arabinofuranose; **FAXG**, α-l-galactopyranosyl-(1→2)-β-d-xylopyranosyl-(1→2)-5-*O*-*trans*-feruloyl-l-arabinofuranose.

Table 1 summarizes the validation parameters for **FA**, **FAX**, and **FAXG**. Because of **FAXG**'s especially sharp peak form, its LOD and LOQ were slightly lower than those of **FAX** and **FA**, but all three compounds displayed LOQs in the single-digit micromolar range. The recoveries for all three compounds were above 93%. The robustness of the method, as shown by its reproducibility over time is illustrated in Figure 3, which depicts the linearity of six separate calibration curves prepared and analyzed 4 months apart, with each calibration curve originating from an individual stock solution. The possibility of quantifying feruloylated side-chains via a ferulic acid standard curve was tested and rejected because the free acid UV response was greater per mole than those of the feruloylated standard compounds (see Supplementary Figures 1–3). This produces the practical limitation of needing to initially isolate the standard compounds for accurate quantification. Nevertheless, the method offers a sensitive, reproducible, accurate possibility for quantification of the major feruloylated arabinoxylan side-chain compounds found in grass cell walls.

**Table 1:** **Calibration equations^a^, correlation coefficients, limits of detection (LOD), limits of quantification (LOQ), and % recovery for FA, FAX, and FAXG**.

	**Range tested (μM)**	**Linear calibration equation**	**Correlation coefficients**	**LOD (μM)**	**LOQ (μM)**	**Recovery (% ± SD)^b^**
FA	10–260	*y* = 26806*x* − 190178	0.9812	1.4	4.2	93.5 ± 5.8
FAX	6–26	*y* = 33194*x* − 29998	0.9607	0.8	2.4	98.7 ± 7.8
FAXG	6–26	*y* = 37649*x* − 37727	0.9813	0.6	1.8	93.4 ± 5.3

a *Calibration equations and correlation coefficients were calculated from three separate calibrations*.

b *n = 3. **FA**, 5*-O-trans-*feruloyl-l-arabinofuranose; **FAX**, β-d-xylopyranosyl-(1→2)-5*-O-*(*trans-*feruloyl)-l-arabinofuranose*.

*FAXG, α-l-galactopyranosyl-(1→2)-β-d-xylopyranosyl-(1→2)-5*-O-trans-*feruloyl-l-arabinofuranose; SD, standard deviation*.

### MS/MS fragmentation

MS/MS fragmentation of the sodium adducts of the reduced standard compounds **FA**, **FAX**, and **FAXG** produced characteristic daughter fragments (see Supplementary Figures 4–6). Fragmentation of the sodium adduct of reduced **FA** ([M + Na]^+^ *m/z* 351) resulted in an *m/z* 333 daughter ion, which represents the sodium adduct of [M − 18]^+^, or the loss of one water molecule. The sodium adduct of reduced **FAX** ([M + Na]^+^ *m/z* 483) yielded one major and one minor daughter ion: *m/z* 351, representing the sodium adduct of the ion created after loss of the xylose unit, and *m/z* 465, representing the sodium adduct of [M − 18]^+^ (loss of one water molecule). MS^2^ fragmentation of the sodium adduct of reduced **FAXG** ([M + Na]^+^ *m/z* 645) produced one major daughter ion: *m/z* 483, representing the sodium adduct of the ion created after loss of the galactose unit. Additionally, two minor daughter ions were observed: *m/z* 627, representing the sodium adduct of [M − 18]^+^ (loss of one water molecule) and *m/z* 351, representing the sodium adduct of the ion created after loss of both the galactose and xylose units.

### Application of feruloylated side-chain profiling method to cereal grain materials

**Figure 4:**
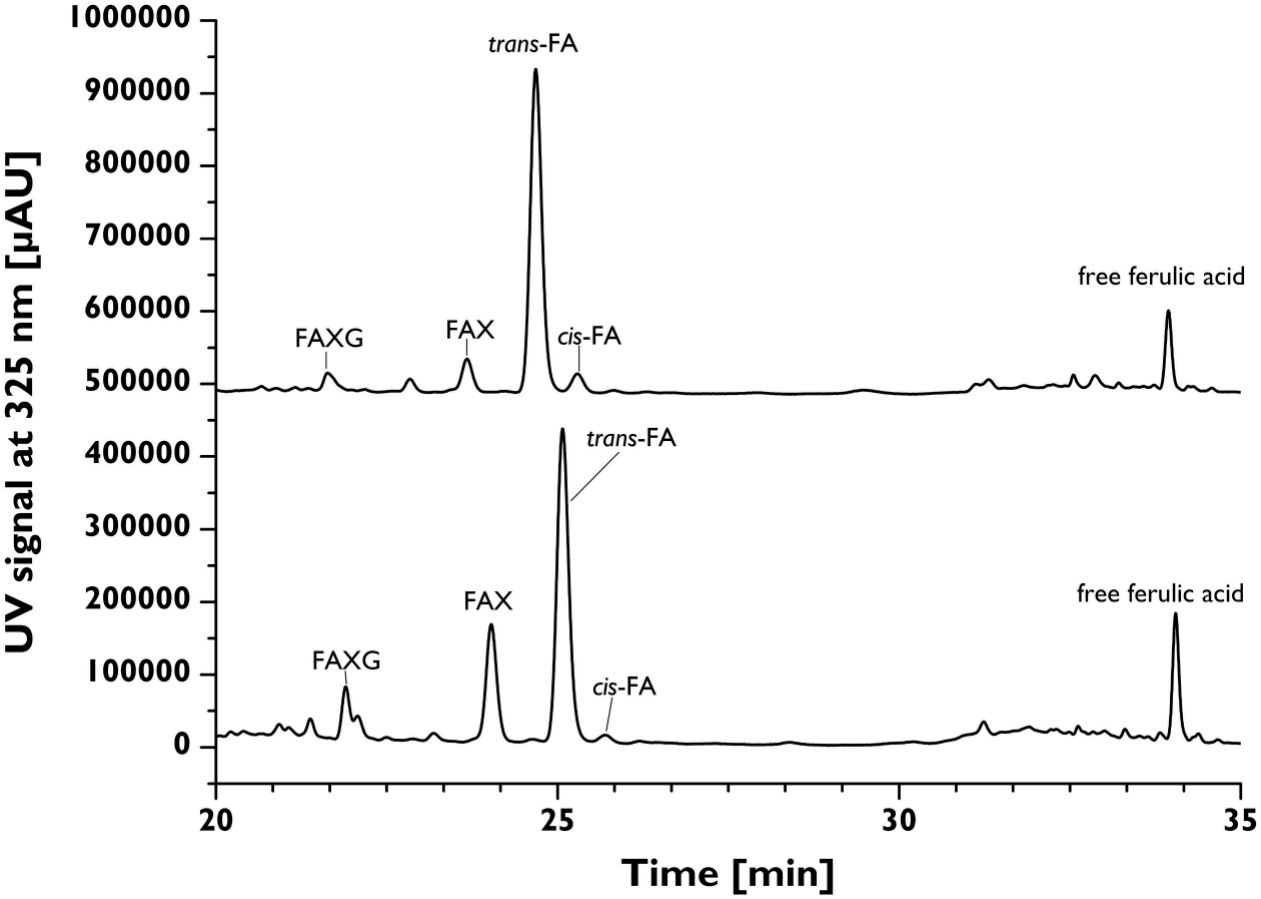
**Chromatographic separation of feruloylated side-chains and free ferulic acid in the TFA hydrolysates from insoluble long-grain rice (upper trace) and popcorn maize (lower trace) fibers following reduction**. **FA**, 5-*O*-*trans*-feruloyl-l-arabinofuranose; **FAX**, β-d-xylopyranosyl-(1→2)-5-*O*-(*trans*-feruloyl)-l-arabinofuranose; **FAXG**, α-l-galactopyranosyl-(1→2)-β-d-xylopyranosyl-(1→2)-5-*O*-*trans*-feruloyl-l-arabinofuranose; TFA, trifluoroacetic acid.

The contents of total esterified ferulic acid (as determined following alkaline hydrolysis with 2M NaOH for 18 h) of the insoluble fiber materials isolated from 12 cereal grains were in the range of about 2.3 (oats) to 33.0 mg/g insoluble fiber (grain maize; Table 2). To study the incorporation of ferulates into arabinoxylan side-chains the developed profiling approach was applied. Running plant cell wall samples required an SPE-C18 clean-up step. Without SPE-C18 clean-up, many hydrolysates formed gels during reduction, which possessed large foam-building capacities during the work-up step and rendered quantitative work very difficult. Following mildly acidic hydrolysis, SPE-18 clean-up, reduction, and LC-DAD/MS separation and detection, the quantitative distribution of the monomeric ferulates into unique feruloylated side-chain profiles was determined for each cereal grain and is shown in Table 3. **FA** was most abundantly liberated from all samples studied, with concentrations ranging between 4.97 (oats) and 100.23 (grain maize) μmol/g insoluble fiber. **FAX** was found in all sample hydrolysates, too, but generally in lower concentrations than **FA** (Table 3). MS/MS fragmentation of the hydrolysates, together with retention time comparison with the **FAXG** standard compound allowed us to both confirm the existence of **FAXG** in all 12 cereal grains screened in this study and quantify this complex feruloylated side-chain in 10 out of 12 cereals (Table 3). For the majority of the cereals, over 70% of their total monomeric ferulates as determined by alkaline hydrolysis were quantitatively captured in their respective side-chain profiles, with values over 90% for several cereals (wheat, spelt, kamut, and wild rice). Values for proso millet, popcorn maize, and intermediate wheatgrass were lower, but still over 50%. Although some free ferulic acid was observed in all TFA hydrolysates (see Figure 4), the quantities were small (<10%) compared to the sum of ferulates captured with the screening method (see Table 3).

**Table 2:** **Total ester-linked ferulic acid contents of insoluble grain fibers^a^**.

**Grain**	**Mean (μg/g insoluble fiber)^b^**	**Standard deviation (μg/g insoluble fiber)**
Barley	6895	242
Grain maize	33109	652
Intermediate wheatgrass	6672	43
Kamut	6141	105
Long grain rice	8991	241
Oats	2294	537
Popcorn maize	30117	456
Proso millet	9840	79
Rye	6240	178
Spelt	8799	408
Wheat	6925	510
Wild rice	2426	223

a *Values determined via alkaline hydrolysis (2M NaOH, 18 h, see Section Materials and Methods for additional details)*.

b *n = 3; values corrected for residual protein and ash in the insoluble fiber material*.

**Table 3:** **Application of the quantitative feruloylated side-chain profiling method to insoluble fibers from whole grains**.

**Grain**	**μmoles FA/g insoluble fiber^a^**	**μmoles FAX/g insoluble fiber^a^**	**μmoles FAXG/g insoluble fiber^a^**	**μmoles free ferulic acid released in TFA hydrolysis/g insoluble fiber^a^**	**FA:FAX ratio**	**sum of ferulates quantified in side-chain profiling method (μmol/g corrected insoluble fiber)^a^**	**% of total ferulates quantified in side-chain profiling method^b^**
Barley	23.74 ± 4.04	2.10 ± 0.41	−^c^	2.29 ± 0.58	11.36	28.13 ± 5.04	78.91 ± 11.19
Grain maize	100.23 ± 2.75	12.70 ± 0.73	3.78 ± 0.28	9.86 ± 1.20	7.90	126.57 ± 4.89	74.27 ± 3.56
Intermediate wheatgrass	20.27 ± 3.77	0.47 ± 0.05	0.14 ± 0.004	1.29 ± 0.36	42.45	22.18 ± 4.15	64.57 ± 12.26
Kamut	26.78 ± 0.91	0.87 ± 0.07	0.21 ± 0.01	2.46 ± 0.15	30.88	30.32 ± 1.03	95.97 ± 5.28
Long grain rice	27.58 ± 4.10	2.21 ± 0.24	0.71 ± 0.03	1.66 ± 0.56	12.45	32.16 ± 4.91	69.30 ± 8.42
Oats	4.97 ± 1.61	1.10 ± 0.41	−^c^	−^d^	4.58	6.06 ± 2.02	50.85 ± 5.87
Popcorn maize	56.75 ± 10.20	20.05 ± 3.51	4.70 ± 0.79	6.54 ± 2.00	2.83	88.07 ± 16.25	56.92 ± 11.37
Proso millet	26.82 ± 1.55	1.25 ± 0.05	0.25 ± 0.005	0.75 ± 0.14	21.47	29.07 ± 1.70	57.35 ± 2.80
Rye	21.59 ± 6.30	1.11 ± 0.25	0.15 ± 0.03	1.84 ± 0.66	19.26	24.69 ± 7.23	76.67 ± 21.39
Spelt	38.17 ± 3.18	0.99 ± 0.06	0.17 ± 0.02	3.09 ± 0.43	38.62	42.43 ± 3.63	94.00 ± 11.87
Wheat	29.82 ± 1.47	0.72 ± 0.09	0.18 ± 0.008	2.43 ± 0.23	41.55	33.14 ± 1.69	93.61 ± 11.98
Wild rice	9.47 ± 1.16^e^	2.39 ± 0.51^e^	0.64 ± 0.08^e^	−^d^	4.47	12.20 ± 1.55^e^	91.86 ± 13.12

a *Average ± standard deviation from triplicate determinations, except where indicated. All values corrected for residual protein and ash*.

b *[(Sum of ferulates quantified from TFA hydrolysis/total ferulates as determined by alkaline hydrolysis) × 100] ± standard deviation*.

c *Detected, but could not be quantified due to co-elution with a matrix component*.

d Detected, but not quantified due to low concentration

e *n = 2*.

***FA**, 5*-O-trans-*feruloyl-l-arabinofuranose; **FAX**, β-d-xylopyranosyl-(1→2)-5*-O-*(*trans-*feruloyl)-l-arabinofuranose; **FAXG**, α-l-galactopyranosyl-(1→2)-β-d-xylopyranosyl-(1→2)-5*-O-trans-*feruloyl-l-arabinofuranose*.

## Discussion

Feruloylated arabinoxylans are an essential structural component of the cell walls of grasses. Feruloylation and subsequent formation of ferulate oligomers was suggested to be an important factor in the regulation of cell wall extensibility (e.g., Azuma et al., 2005) and building first line defense mechanisms of the plant against invading pathogens (e.g., Santiago and Malvar, 2010). Feruloylated arabinoxylans form a substantial portion of the health-promoting dietary fiber complex in human diets containing whole grains, where they may produce both prebiotic and (potentially) antioxidative health benefits (Broekaert et al., 2011). Additionally, recalcitrance of biomaterials during lignocellulosic biofuel production and the digestibility of both stover and grain-based animal diets are substantially influenced by (di)feruloylated arabinoxylans. Mounting evidence points toward the possibility that feruloylated arabinoxylans' effects, particularly on enzymatic digestibility, are mediated not only by ferulate dimers/higher oligomers' crosslinking of cell wall polymers, but also by the degree and complexity of their monomeric feruloylated side-chain substituents. In isolated enzyme studies, many (but not all) arabinofuranosidases are unable to cleave feruloylated arabinose from the xylan backbone (Wood and McCrae, 1996; Luonteri et al., 1999; Remond et al., 2008). Structural characterization of feruloylated maize arabinoxylo-oligosaccharides resistant to mild acid pretreatment followed by enzymatic saccharification with an enzyme cocktail showed that most of these oligosaccharides contained the **FAXG** or **FAX** structural moiety (Appeldoorn et al., 2013). In addition, feruloylated oligosaccharides represented 39% of the enzyme-resistant oligosaccharides. Yang et al. (2013) found differing short chain fatty acid profiles from *in vitro* fermentation with human fecal microbiota of autohydrolysates from wheat and maize brans and suggested that these differences may have been caused by the materials' divergent feruloylated side-chain profiles. Feruloylation of arabinoxylo-oligosaccharides limited their fermentability by human colon microbiota compared to non-feruloylated arabinoxylo-oligosaccharides (Snelders et al., 2014). In some contexts, such as lignocellulosic biofuel production, slower rates of enzymatic digestion or recalcitrance to digestion are an economical and processing drawback, although in other situations, this may be beneficial. For example, in fermentation of dietary fiber polysaccharides by the human gut microflora, a more slowly digestible arabinoxylan represents a potential delivery source of fermentable carbohydrates to the distal colon, which may be a boon to colon health.

Numerous methods for the determination of esterified ferulates in plant materials exist; however, only a few approaches aim to discriminate between different ferulate populations in plant materials and/or to localize the ferulates' position in cell wall polymers. Vaidyanathan and Bunzel (2012) presented a method capable of dividing a plant material's total monomeric ferulates into four populations: ester-linked to insoluble fibers, ester-linked to soluble fibers, ester-linked to oligosaccharides, and free ferulic acid. Philippe et al. (2007) created an elegant method for investigating local deposits of **FA** within the cell using a polyclonal antibody. Until now, however, a quantitative comparison of the feruloylated arabinoxylan side-chain profiles from varying plant cell wall sources has not been described. To date, information about ferulate incorporation into more complex arabinoxylan side-chains was merely based on NMR spectroscopic characterization of preparatively isolated feruloylated arabinoxylan fragments (e.g., Saulnier et al., 1995 and Allerdings et al., 2006), an approach that works well for plant materials rich in a specific structural element but is extremely laborious for plant materials low in a specific structural unit. Thus, we developed an efficient quantitative screening method for feruloylated side-chain substituents of arabinoxylans in plant cell walls following a mildly acidic hydrolysis, SPE-C18 clean-up, reduction, and LC-DAD/MS separation and detection. Although the method is not quantitative in the absolute sense (the reported side-chain concentrations should not be construed as the absolute total in the material, but rather the portion released under the hydrolysis conditions), the method offers the possibility for a quantitative *comparison* of feruloylated side-chain profiles in different cell wall materials.

Quantitative liberation of feruloylated arabinoxylan side-chains is the major shortcoming of our approach. Enzymatic approaches are not feasible because the studied structural elements hinder enzymatic cleavage, as detailed above. Chemical approaches are often only semi-specific and need to be optimized for liberation versus destruction of specific building blocks of polymers. The mildly acidic hydrolysis method applied in our study was optimized earlier by Saulnier et al. (1995) for semi-specific release of feruloylated arabinoxylan side-chains and capitalizes on the greater acid lability of furanosidic compared to pyranosidic linkages. The recommended hydrolysis conditions (50 mM TFA, 100°C, 2 h), which were used in this study, simultaneously balance the maximized cleavage of arabinofuranose-based side-chains with minimal degradation of ferulate ester bonds. However, the non-esterified ferulic acid detected in the hydrolysates (Table 3) is clearly a hydrolysis artifact, because any free ferulic acid naturally occurring in the plant materials is removed by washing steps at the end of the insoluble fiber isolation.

Keeping this limitation in mind, distinctive patterns were revealed in the cereals' profiles. Generally, the side-chain profiling method mirrored the total ferulate (alkaline hydrolysis) data: grains with higher total ferulate contents (for example, popcorn maize and grain maize) showed the highest concentration of side-chains and grains with lower total ferulate contents (such as oats and wild rice) produced the lowest concentration of side-chains. Interestingly, total side-chain concentration was not a direct predictor of the complexity of the side-chain profile, as determined by the **FA:FAX** ratio. For example, although oats' total feruloylated side-chain concentration was the lowest of the 12 grains screened in this study, its **FA**:**FAX** ratio was the third-most complex, topped only by wild rice and popcorn maize. The simplest side-chain profile was produced by intermediate wheat grass, a perennial grain already reported to possess simple, relatively unsubstituted arabinoxylans (Schendel et al., 2015). The three tested *Triticum* species (wheat, spelt, and kamut) clustered together with an extremely low level of side-chain complexity, producing **FA:FAX** ratios between 30 and 40. Additionally, the amount of total monomeric ferulates captured in their side-chain profiles was over 90%, indicating that nearly all of the monomeric ferulates in these species are bound to arabinoxylans in the form of the simplest possible feruloylated side-chain, **FA**. The **FA:FAX** ratio of 2.83 from popcorn maize, on the other hand, represents a high level of side-chain complexity: every third feruloylated side-chain substituent in popcorn arabinoxylans is **FAX**. Compared to popcorn maize, grain maize had a larger concentration of total side-chains, but a simpler complexity profile. Barley and long grain rice produced similar profiles, with moderate levels of both **FA** and **FAX**. As already mentioned, oats, but also wild rice, resulted in the surprising profiles of low absolute total side-chain levels, but high relative values of side-chain complexity. Another surprising result of our screening was the discovery of low levels of **FAXG**, which had previously only been described in maize (Allerdings et al., 2006), in all tested cereals. Although the sheer quantity of **FAXG** in maize grain cell walls is clearly higher than in other cereals, its biosynthesis in grain cell walls appears to be ubiquitous throughout the Poaceae family.

For most of the screened cereals, the side-chain profiling method quantified more than 70% of their total ester-linked monomeric ferulate populations, with intermediate wheatgrass, oats, popcorn maize, and proso millet standing as exceptions. It is possible that biosynthesis in these cereals directs a larger portion of alkali-hydrolysable monomeric ferulates into other structural depots resistant to the mild TFA hydrolysis conditions in this study, such as ester-linked to lignin or other polyphenolic or polyaliphatic compounds such suberin-type polymers. Two-dimensional NMR analysis of the residues remaining after TFA hydrolysis could serve as a starting point for investigating this possibility.

Another aspect that needs to be studied in future is whether or not the complexity of the feruloylated side-chains has an impact on peroxidase- and laccase-mediated diferulate formation. It can be hypothesized that simple feruloylated side-chains are more heavily involved in cross-link formation than sterically complex, hindered feruloylated arabinoxylan side-chains.

## Conclusion

The developed method enables the quantitative comparison of feruloylated side-chain profiles between different cell wall materials, opening new possibilities for determining arabinoxylan structure-function relationships in the future. We found that feruloylated arabinoxylans from cereal grains differed substantially from each other in some aspects, such as **FA:FAX** ratio. We also confirmed the presence of **FAXG** in all tested grains.

## Author contributions

MB and RS designed the research, RS and MM conducted the experiments, RS and MM analyzed the data results. RS and MB wrote the manuscript. All authors read and approved the final manuscript.

### Conflict of interest statement

The authors declare that the research was conducted in the absence of any commercial or financial relationships that could be construed as a potential conflict of interest.

## Abbreviations

FA: 5-*O*-*trans*-feruloyl-l-arabinofuranose
FAX: β-d-xylopyranosyl-(1→2)-5-*O*-(*trans*-feruloyl)-l-arabinofuranose
FAXG: α-l-galactopyranosyl-(1→2)-β-d-xylopyranosyl-(1→2)-5-*O*-*trans*-feruloyl-l-arabinofuranose
FAXGG: α-d-galactopyranosyl-(1→3)-α-l-galactopyranosyl-(1→2)-β-d-xylopyranosyl-(1→2)-5-*O*-*trans*-feruloyl-l-arabinofuranose
FAXGX: α-d-xylopyranosyl-(1→3)-α-l-galactopyranosyl-(1→2)-β-d-xylopyranosyl-(1→2)-5-*O*-*trans*-feruloyl-l-arabinofuranose
LOD: limit of detection
LOQ: limit of quantification
SPE: solid phase extraction
TFA: trifluoroacetic acid.
