# Supplementary material for: Quantitative Profiling of Feruloylated Arabinoxylan Side-Chains from Graminaceous Cell Walls
Source: Front Plant Sci. 2016 Jan 14;6:1249. doi: 10.3389/fpls.2015.01249 (PMC4712305; doi:10.3389/fpls.2015.01249)
Supplement: Supplementary file 1 [file Image1.PDF]

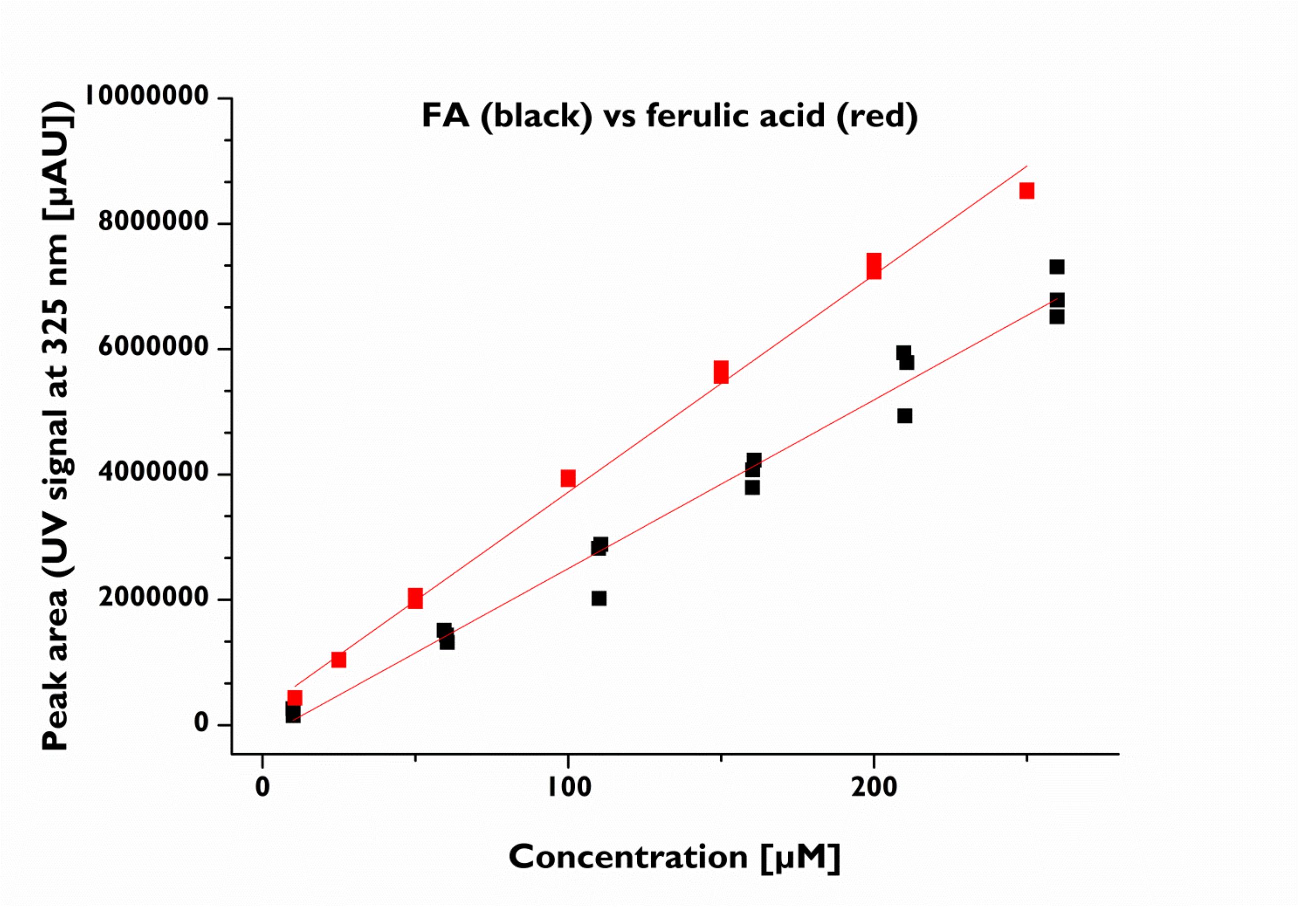


**Supplementary Figure 1.** Linear response of **FA** (black) compared to ferulic acid (red). **FA**: 5-*O*-*trans*-feruloyl-L-arabinofuranose

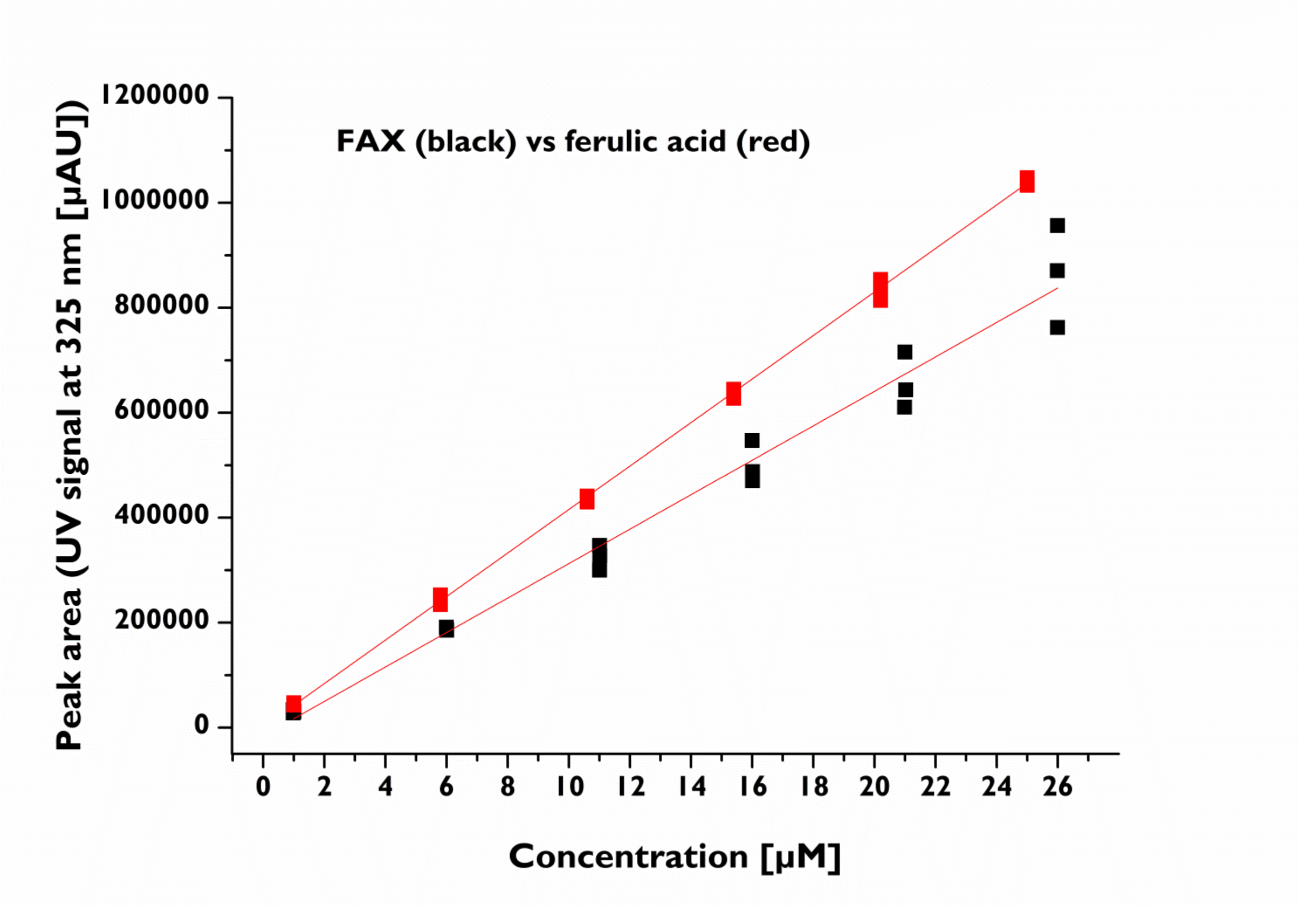


**Supplementary Figure 2.** Linear response of **FAX** (black) compared to ferulic acid (red). **FAX**: β-D-xylopyranosyl-(1→2)-5-*O*-(*trans*-feruloyl)-arabinofuranose

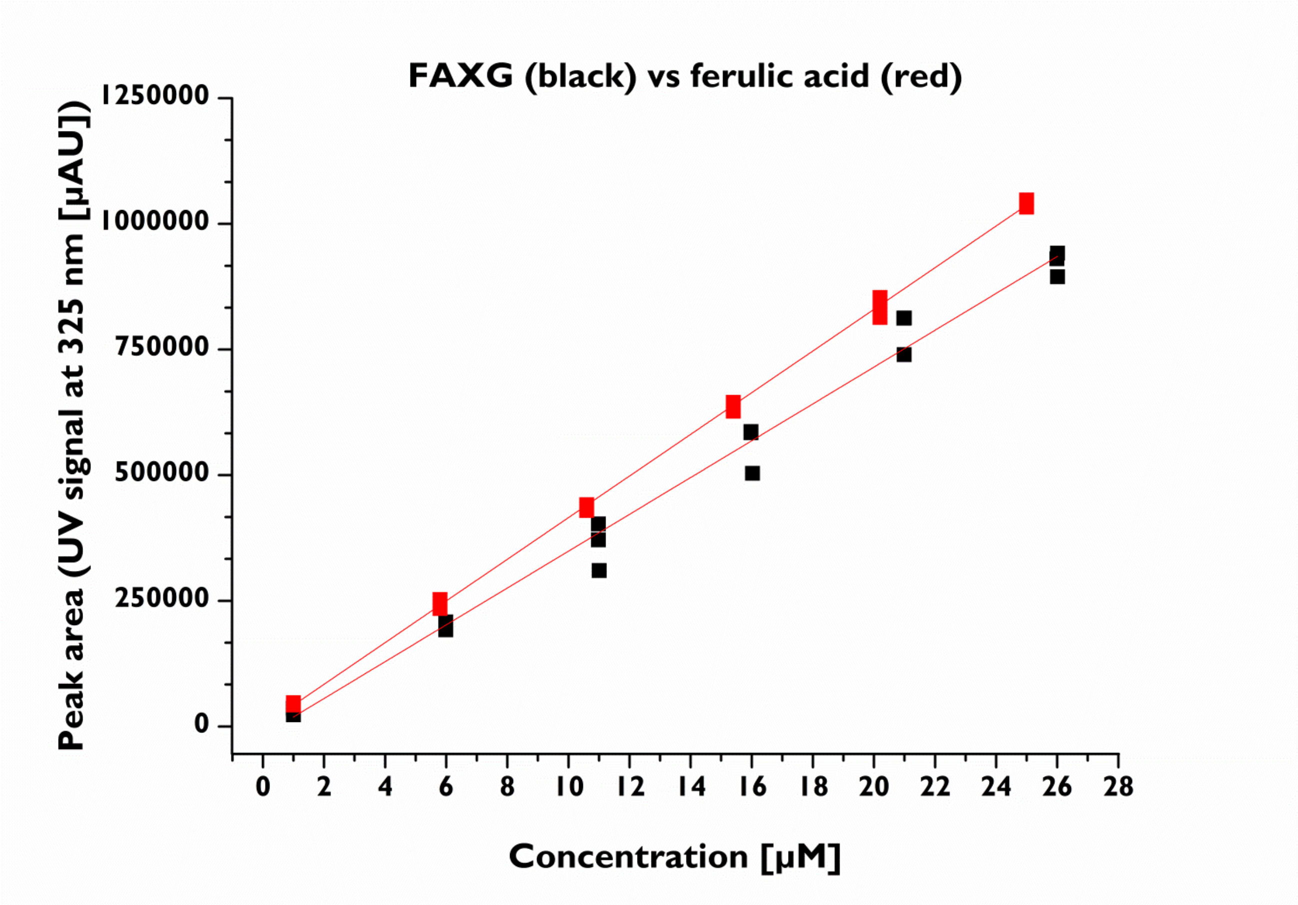


**Supplementary Figure 3.** Linear response of **FAXG** (black) compared to ferulic acid (red). **FAXG**: α-L-galactopyranosyl-(1→2)-β-D-xylopyranosyl-(1→2)-5-*O*-*trans*-feruloyl-L-arabinofuranose

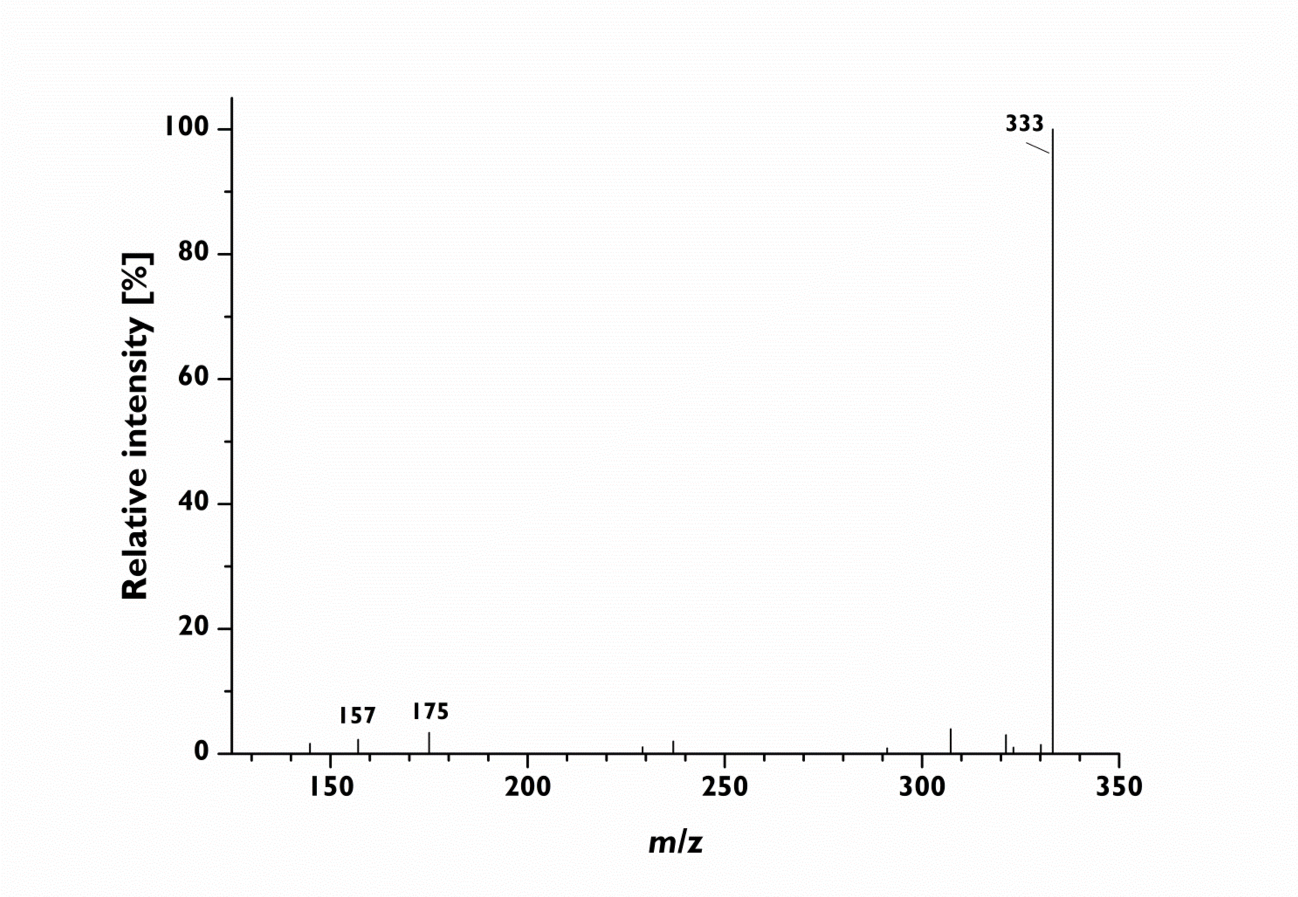


**Supplementary Figure 4.** MS/MS spectrum of the sodium adduct of reduced **FA** ($[M + Na]^+$ *m/z* 351). **FA**: 5-*O*-*trans*-feruloyl-L-arabinofuranose

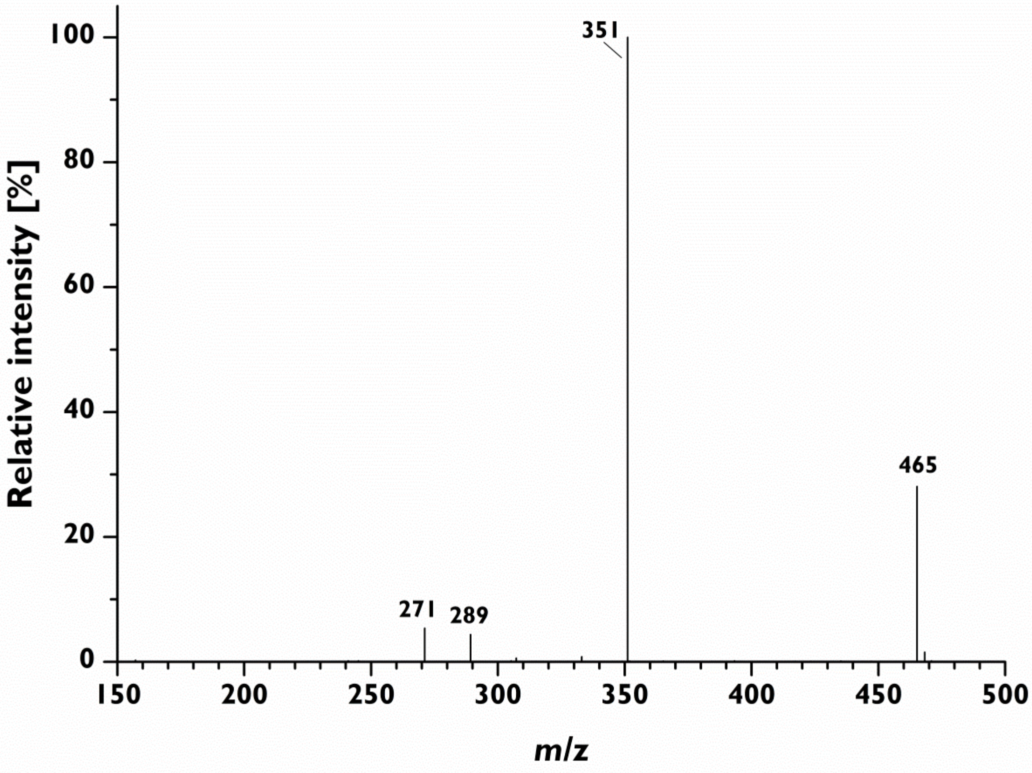


**Supplementary Figure 5.** MS/MS spectrum of the sodium adduct of reduced **FAX** ($[M + Na]^+$ *m/z* 483).
**FAX**: β-D-xylopyranosyl-(1→2)-5-*O*-(*trans*-feruloyl)-arabinofuranose

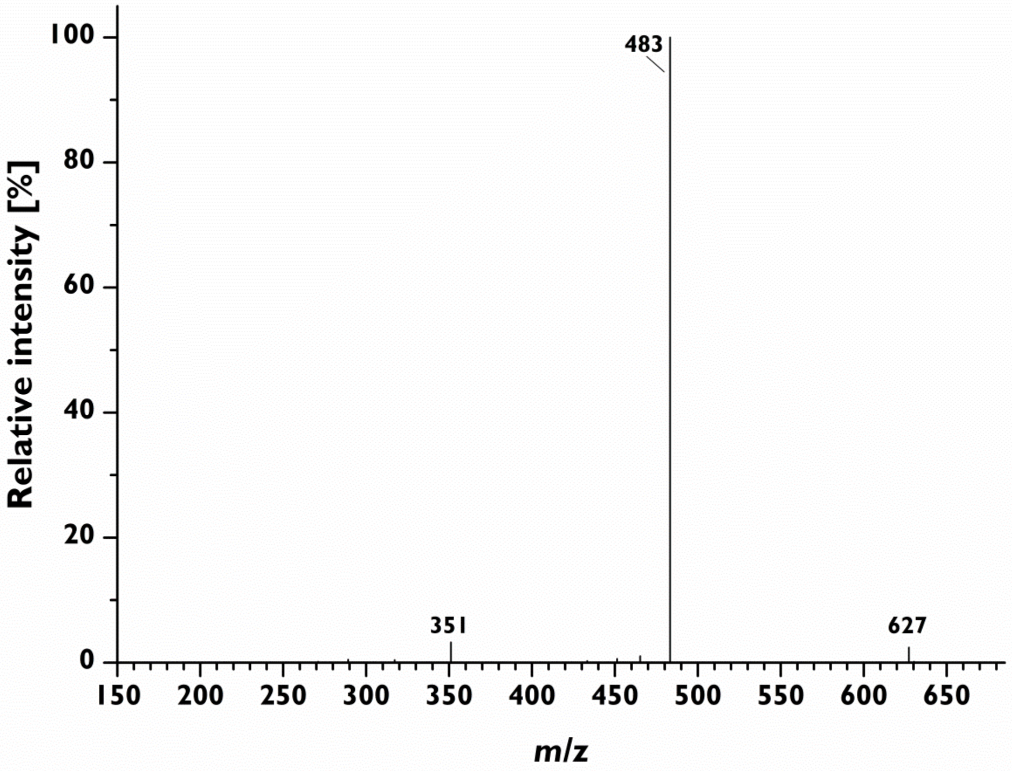


**Supplementary Figure 6.** MS/MS spectrum of the sodium adduct of reduced **FAXG** ($[M + Na]^+$ *m/z* 645).
**FAXG**: α-L-galactopyranosyl-(1→2)-β-D-xylopyranosyl-(1→2)-5-*O*-*trans*-feruloyl-L-arabinofuranose
